# Comparison of Ultrasound-Guided Fine-Needle Cytology Quality in Thyroid Nodules with 22-, 23-, and 25-Gauge Needles

**DOI:** 10.1155/2021/5544921

**Published:** 2021-06-07

**Authors:** YiJie Dong, LiLi Gao, Yang Sui, MinJing Mao, WeiWei Zhan, JianQiao Zhou

**Affiliations:** ^1^Department of Ultrasound, Ruijin Hospital, Shanghai Jiaotong University Medical School, Shanghai 200025, China; ^2^Department of Pathology, Ruijin Hospital, Shanghai Jiaotong University Medical School, Shanghai 200025, China; ^3^Department of Ultrasound, First Affiliated Hospital of Bengbu Medical College, Bengbu 233000, China; ^4^Department of Laboratory, Ruijin Hospital, Shanghai Jiaotong University Medical School, Shanghai 200025, China

## Abstract

**Objective:**

To compare the cytology quality of ultrasound-guided fine-needle biopsy in thyroid nodules with 22-, 23-, and 25-gauge (G) needles prospectively.

**Methods:**

A total of 240 consecutive nodules underwent ultrasound-guided fine-needle aspiration (USG-FNA) and 240 nodules underwent ultrasound-guided fine-needle capillary (USG-FNC) were included in this prospective study from October 2014 to February 2016. Each nodule was sampled using 22 G, 23 G, and 25 G needle according to designed orders, and 1240 smears were finally obtained. Cytology quality was scored by a cytologist blinded to needle selection.

**Results:**

In USG-FNA, the average scores and standard deviations were 5.50 ± 2.87 for 25 G needles, 4.82 ± 2.95 for 23 G needles, and 5.19 ± 2.81 for 22 G needles. In USG-FNC, the average scores and standard deviations of each group were 5.12 ± 2.69 for 25 G, 4.60 ± 2.90 for 23 G, and 4.90 ± 2.90 for 22 G needles. The specimen quality scores of 25 G group were significantly higher than that of 23 G group (*P* < 0.017) in both USG-FNA and USG-FNC. However, the differences were not statistically significant in nondiagnostic rate using different gauge of needles (*P* > 0.017 for all).

**Conclusions:**

25 G needles obtained the highest scores of sample quality in thyroid FNA and FNC comparing with 22 G and 23 G needles. 25 G needle should be first choice of thyroid FNA and FNC in routine work.

## 1. Introduction

There is an increasing population of thyroid nodule patients in the world. About 30%~60% of the adult harbors at least one thyroid nodule. Ultrasound-guided fine needle biopsy (USG-FNB) is a simple and fast technique, which is recognized as the gold standard of preoperative diagnosis in thyroid cancers [1–4]. Cytology results of USG-FNB in thyroid nodules can be affected by many factors including punctuation method (ultrasound-guided fine needle aspiration and ultrasound-guided fine needle capillary, USG-FNA and USG-FNC), needle gauges and types, passes of punctuation, and sample preparation [2, 5, 6]. In the procedure of USG-FNB, the choice of which gauge of needle to use is the first issue to be addressed. Previous studies reported that 21-27-gauge (G) needles can be used in USG-FNA and USG-FNC [7–10]. And 22-25-gauge needles were most commonly used in the majority of reported studies [1, 2, 10]. However, it had not been evaluated how needle gauge affected cytology specimen quality in routine work of USG-FNA and USG-FNC.

Therefore, the purpose of this study was to prospectively compare the quality of cytology specimens of thyroid nodules obtained by 22 G, 23 G, and 25 G needles in USG-FNA and USG-FNC.

## 2. Material and Methods

### 2.1. Patients

This prospective study was approved by the Institutional Review Board and Ethics Committee, and all the patients had signed informed consent before they underwent ultrasound-guided fine needle biopsy. From October 2014 to February 2016, a total of 480 nodules in 437 consecutive outpatients underwent USG-FNA and USG-FNC in our department. There were 240 nodules from 221 consecutive patients underwent USG-FNA. Forty-nine were male, and 172 were female. The average age was 45.52 ± 13.06 years old (range 23-76 years old). Another 240 nodules from 216 consecutive patients underwent USG-FNC. Fifty-two were male, and 164 were female. The average age was 46.26 ± 13.71 years old (range 22-92 years old).

### 2.2. Methods

#### 2.2.1. USG-FNA and USG-FNC Procedures

**Figure 1:**
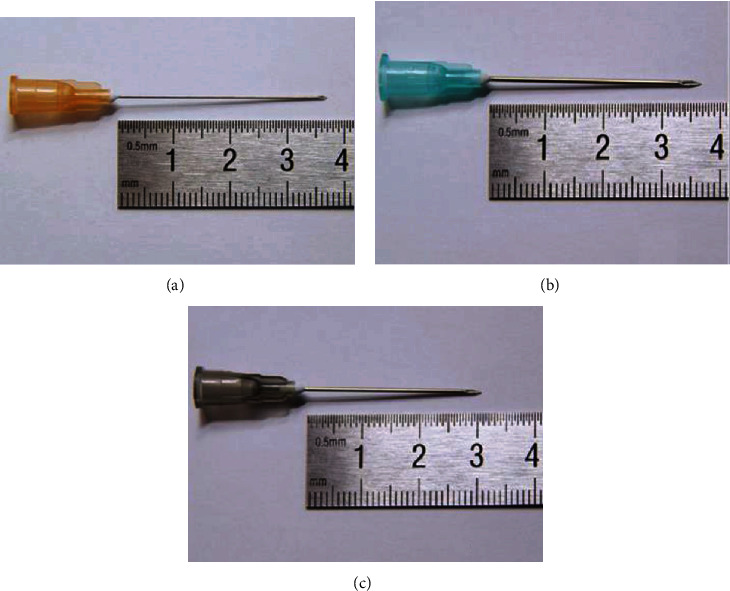
Fine needles used in the present study: 25 G with length of 37 mm (a), 23 G with length of 37 mm (b), and 22 G with length of 31 mm (c).

USG-FNA and USG-FNC were performed by two radiologists (experienced 7 years and 15 years in intervention) using two commercially available ultrasound scanners (Siemens S2000, German; Samsung RS80A, Korea) equipped with high-resolution probes (7-14 MHz). Target nodules were selected according to the guidelines of the European Thyroid Association (ETA) and the American Thyroid Association for the management of thyroid nodules and differentiated thyroid cancer [1, 11]. Each nodule was sampled three passes using a 25 G fine needle with length of 37 mm, a 23 G fine needle with length of 37 mm, and a 22 G fine needle with length of 31 mm (Figure 1). Punctuation procedures were performed without local anesthesia. Adjust the ultrasound probe position so that the ultrasound beam is parallel to the fine-needle to guide punctuation and biopsy in real time. In USG-FNA procedure, needle was attached a 5 ml syringe. When the fine-needle reached the target place of the nodule, the operator moved the needle back and forth with suction applied until the sample material were aspirated into the hub of the needle. In USG-FNC procedure, no syringe was attached, and no suction was applied. The operator only moved the needle tip back and forth rapidly within the nodule until sample material rises into the hub. The obtained material was smeared on glass slides and sent for cytopathological examination immediately.

**Figure 2:**
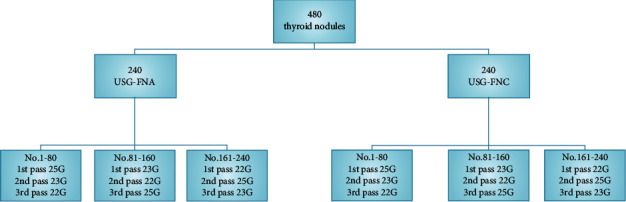
Protocol of USG-FNA and USG-FNC using 22 G, 23 G, and 25 G needles.

In order to minimize the selecting bias, each nodule was sampled 3 passes of USG-FNA by 22-25 G needles according to following sequence: consecutive nodules from No. 1 to No. 80 were aspirated in the order of 25 G, 23 G, and 22 G needle; nodules from No. 81 to No. 160 were aspirated in the order of 23 G, 22 G, and 25 G; nodules from No. 161 to No. 240 were aspirated in the order of 22 G, 25 G, and 23 G (Figure 2). Each pass applied to one smear. Smears were marked 1, 2, and 3 representing the sequence of passes. Ultrasound examination was performed 20 minutes after US-guided FNA procedure to assess whether there was complication occurred. The same protocol was used in USG-FNC procedure. A total of 720 smears by USG-FNA and 720 smears by USG-FNC were finally obtained (one smear per punctuation).

#### 2.2.2. Cytology Interpretation

Extrusions of sample material obtained by FNA or FNC were placed on glass slides. Smears were fixed in alcohol and stained with hematoxylin and eosin (H&E staining). One cytologist (experienced 38 years, blinded to methods of punctuation) assessed each smear based on the scoring system reported by Haddadi [12]. There were 4 parameters including background clot or blood, number of obtained cells, preserved tissue architecture, and cellular degeneration in this scoring system. Each parameter had a score between 0 and 2 according to different quality of the smear. The total scores of the 4 parameters were categorized as follows: 0-2 as inadequate for diagnosis, 3-5 as adequate for diagnosis, and 6-8 as superior for diagnosis (Table 1). The final results of cytology was reported using the Bethesda criteria as follows: nondiagnostic, benign, indeterminate (including atypia of undetermined significance, follicular lesion of undetermined significance, suspicious for a follicular neoplasm, and follicular neoplasm), suspicious for malignancy, and malignancy [13, 14].

### 2.3. Statistic Methods

Clinical data including age, gender, location of nodules, and cytological results were recorded in Microsoft Excel software. Cumulative scores of 3 groups of USG-FNA using 22 G, 23 G, and 25 G were analysed with a commercially available software SAS statistical software package (version 9.4). The general linear model (GLM) and repeated measure analyses of variance (ANOVA) were used to compare the average scores of specimens in 3 groups. Nondiagnostic rate among 22 G, 23 G, and 25 G groups were compared using Chi-square tests. Differences in measurement data were tested using *t*-test. Chi-square or Kruskal Wallis test was used to evaluate the adequacy of the smears (SPSS version 17.0; SPSS Inc., Chicago, Ill). For the multiple pairwise comparison, a *P* value less than the Bonferroni-corrected significance value of 0.05/3 = 0.017 was considered to indicate a statistically significance. Otherwise, *P* < 0.05 was considered to indicate a significant difference.

## 3. Results

The diameter of 240 nodules in group of USG-FNA and of 240 nodules in USG-FNC was 10.9 ± 6.9 mm (range 2.8-52 mm) and 10.5 ± 7.5 mm (range 2.9-46 mm), respectively (*P* = 0.562). No severe hemorrhage and other complications were found in this study. The Bethesda category for cytopathological diagnosis was shown in Table 2. After completing three passes of sampling using 22 G, 23 G, and 25 G needles, respectively, sufficient samples could be obtained in the vast majority of nodules for cytological diagnosis. The overall nondiagnostic rate was only 1.25% (3/240) both in the USG-FNA and USG-FNC group.

### 3.1. Quality of USG-FNA Specimens Using 22 G, 23 G, and 25 G Needles

In USG-FNA group, there were statistically significant differences of the adequacy of the smears among 25 G, 23 G, and 22 G needles (*P* = 0.027) (Table 3). Specifically, the inadequate (0-2 points) rate was 14.17% for 25 G needle, 19.58% for 23 G needle, and 15.42% for 22 G needle, respectively. No significant differences were found in multiple pairwise comparisons for inadequate rate in these three groups (*P* > 0.017 for all). Similarly, no significant differences in adequate (3-5 points) rate were found (*P* > 0.017 for all). The excellent quality (6-8 points) rate was 52.92%, 40.83%, and 45.42% for 25 G, 23 G, and 22 G needles, respectively. Compared with 23 G needle, 25 G needle obtained a higher rate of excellent quality specimens (*P* = 0.008). However, similar results did not appear in the comparison of 25 G and 22 G, nor in the comparison of 23 G and 22 G (*P* > 0.017 for both).

**Figure 3:**
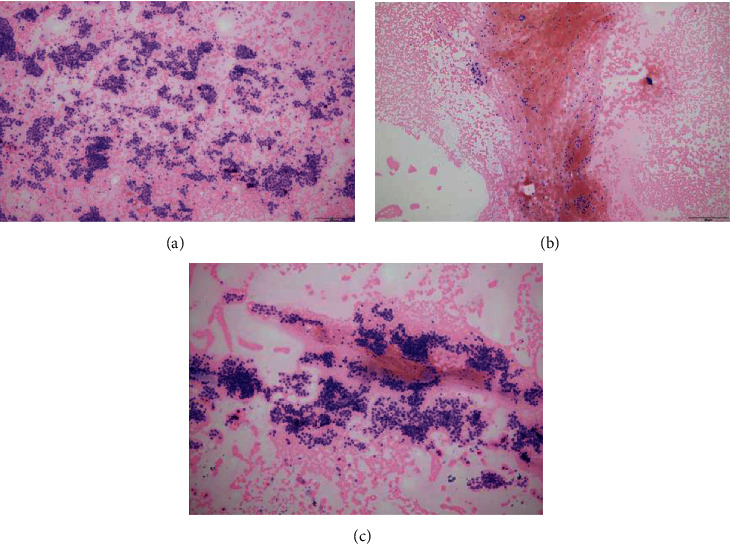
HE-stained cytological smears of thyroid nodules obtained by FNA (10x magnification). Cytological smear of a nodule obtained by 25-gauge needle, with clean background and less blood cells, scattered and abundant of tissues with clear structures, and ordered cells arrangement (a); smear of the same nodule obtained by 23-gauge needle, with abundant blood cell and clot and fewer follicular epithelial cells (b); smear of the same nodule obtained by 22-gauge needle, with disordered background, abundant blood cells and fewer clot, gathered tissues, and basically, clear structures (c).

The scores of the total specimen quality and of each cytological parameter assessed under microscopy in FNA group were listed in Table 4. The total score obtained by 25 G needle was the highest (5.50 ± 2.87), followed by 22 G needle (5.19 ± 2.81) and 23 G needle (4.82 ± 2.95). The scores of each of the four cytological parameters also reflected such trend, that is, the score obtained by 25 G was the highest, followed by 22 G, and the lowest was by 23 G (Figure 3). The scores of the total specimen quality and the four parameters of 25 G group were significantly higher than those of 23 G group (*P* < 0.017 for all). However, there is no significance of scores between the groups of 25 G and 22 G, as well as between 22 G and 23 G (*P* > 0.017 for all).

### 3.2. Quality of USG-FNC Specimens Using 22 G, 23 G, and 25 G Needles

In USG-FNC group, there were no statistically significant differences of the adequacy of the smears among 25 G, 23 G, and 22 G needles (*P* = 0.192) (Table 3). Moreover, no significant differences were found in multiple pairwise comparisons for inadequate rate, for adequate rate, and for excellent quality rate in these three groups (*P* > 0.017 for all).

**Figure 4:**
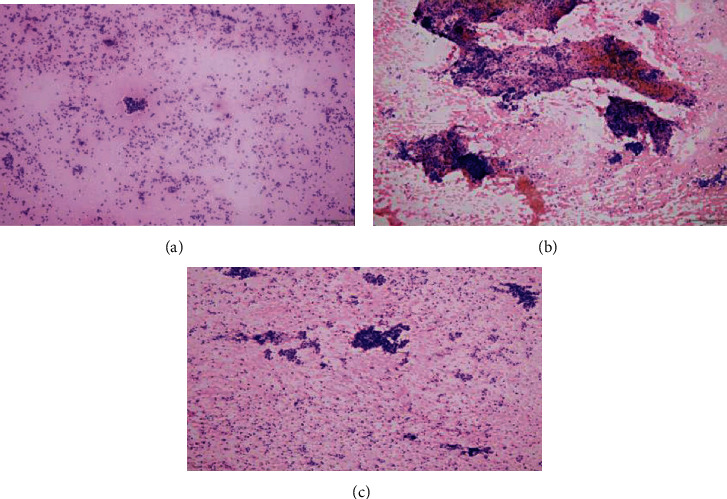
HE-stained cytological smears of thyroid nodules obtained by FNC (10x magnification). Cytological smear of a nodule obtained by 25-gauge needle, with clean background, scattered but plenty of tissues, clear structures, and follicular epithelial cells (a); smear of the same nodule obtained by 23-gauge needle, with disordered background, plenty of blood cells, and partially overlapped cells (b); smear of the same nodule obtained by 22-gauge needle, with cleaner background, less blood clots, and basically, clear structures (c).

Table 4 showed the scores of the total specimen quality and of each cytological parameter in FNC group. The total score was 5.12 ± 2.69 for 25 G, followed by 4.90 ± 2.90 for 22 G needles, and 4.60 ± 2.90 for 23 G. The scores of each of the four cytological parameters also reflected such trend. The results of statistical analysis were similar to those of FNA group, that is, the cytology scores of the total specimen quality and each cytological parameter of the 25 G group were significantly higher than those of the 23 G group (*P* < 0.017 for all), and there was no significance between the groups of 25 G and 22 G, as well as 22 G and 23 G (*P* > 0.017 for all) (Figure 4).

## 4. Discussion

The selection of needle is an essential technical element of fine needle biopsy. Needle gauge is an important factor that is affecting results of both conventional smear cytology and liquid-based cytology [4, 7, 9, 15]. In this paper, we objectively investigate the effect of needle gauge on specimen quality during USG-FNB, including FNC and FNA.

The adequacy of smear was an important basis for choosing the right puncture needle. The scoring system proposed by Mair et al. [16], based on 100 lesions from different body sites, was one of the most widely adopted scores to compare the smear quality. Scoring system for thyroid has also been developed [12, 17]. Our study used the thyroid scoring system proposed by Haddadi-Nezhad et al. [12], in which background clot or blood, number of obtained cells, preserved tissue architecture, and cellular degeneration of the smear were evaluated.

In this prospective study, cytological smear samples obtained by 22 G, 23 G and 25 G fine needles were compared, and the factors such as patients and nodule characteristics were excluded due to the three different needles were sampled in the same nodule. We found that both in USG-FNA and USG-FNC groups, the scores of the total specimen quality and of each cytological parameter obtained by 25 G needle were the highest, followed by 22 G needle and 23 G needle. There were significant differences between 25 G needle and 23 G needle groups (*P* < 0.017 for all). However, there were no significances between the groups of 25 G and 22 G, as well as between 22 G and 23 G (*P* > 0.017 for all). Our results revealed that in general, samples obtained by 25 G needle contained less blood cells and clot stained and provided well preserved tissue architecture, as well as larger quantities of cells with minimal cellular degeneration. This was consistent with the findings of Degirmenci et al. [18] who compared usefulness of 20 G and 24 G needles in USG biopsy in thyroid nodules and found that more material was obtained using thinner needles (24 G), while bloodstained material was more frequently seen in aspiration with 20 G needles. However, the study performed by Tangpricha et al. [7] indicated that larger needles (21 G) provide more cellularity than thinner ones (25 G) by FNA.

Interestingly, 22 G needle was larger in caliber than 23 G needle, but both in the FNA and FNC groups, specimen scores obtained by the 22 G needle were higher than those by 23 G needle, although there was no statistical difference. The reason for this phenomenon appeared to be more difficult to explain and therefore required more in-depth research in the future. We noted that the 22 G needle was 31 mm long, while the 23 G needle was 37 mm long, but there was no reason to speculate that the 16% length difference reduced the score of the 23 G needle, because the 25 G needle with the length of 37 mm received the highest scores. However, in theory, longer needles may be prone to more blood cell clotting.

In both the FNA group and FNC group, there were no significant differences in the proportion of inadequate samples (0-2 points) among needles with different calibers, nor in the proportion of adequate samples (3-5 points). However, 25 G needle obtained a higher proportion of excellent quality specimens (6-8 points) as compared with 23 G needle in the FNA group. Our results were similar to previous studies. Regarding the unsatisfactory rates, Tanaka et al. found that there was no statistical difference between 22 G and 25 G needles [19]. In terms of adequacy, there was no statistically significant difference between 21 G and 27 G needles [8], between 22 G and 27 G needles [20], and among 23 G, 25 G, and 27 G needles [21]. Therefore, more cellular specimens did not necessarily result in increased diagnostic accuracy. Different gauge of needles may not affect diagnostic accuracy [7]. In technique aspect, the physical characteristics of three needles were different. The length of 22 G, 23 G and 25 G needles used in our study were 31 mm, 37 mm, and 37 mm, respectively. Therefore, compared with the 22 G needle, the 25 G and 23 G needles have the advantage of being able to sample deep thyroid nodules in obese patients. However, because of its slenderness, 25 G needle was soft and easy to bend; sometimes, it may lead to sampling difficulties, which required higher skills for doctors. Besides, thinner and longer needles were easier to be blocked by cells and tissues, making the smearing process more time-consuming. The 22 G needle was relatively thick and short, so it was hard and not easy to bend, which was convenient for adjusting the puncture direction at any time. It was recommended using 22 G needles for markedly calcified nodules because 25 G needles bend more easily in such nodules [19]. Moreover, the 22 G needle has better visibility on the ultrasound image.

From the perspective of clinical use, thinner needles were recommended as a priority in order to reduce complications, as studies have found that larger needle injured thyroid gland severer than thinner one [7, 8]. Moreover, smaller needles used in punctuation were more tolerable psychologically for patients, although previous studies found that there was no difference in pain scale of punctuation using different caliber of needle [2, 15, 22].

There were some limitations of this study. First, only one cytologist assessed the smears which might lead to subjectivity. Second, only H&E staining was used to assess the cytology quality, but the commonly used Papanicolaou or Romanowsky stainings were not applied. Third, pathological results were unable to provide because of outpatients were selected in this prospective study.

In conclusion, based on this prospective study including 240 consecutive nodules, it can be found that 25 G needles obtained the highest scores of sample quality in thyroid FNA and FNC comparing with 22 G and 23 G needles. We suggested that 25 G needle should be the first choice of thyroid FNA and FNC in routine work.

## Figures and Tables

**Table 1:** Scoring system for specimen quality analysis for thyroid nodules.

Smear parameters and quality description	Scores
Background of blood or clot	
Excessive: nondiagnosis	0
Intermediate: diagnosis possible	1
Minimal: diagnosed easily	2
Number of obtained cells	
Minimal: nondiagnosis	0
Intermediate: diagnosis possible	1
Abundant: diagnosed easily	2
Preserved tissue architecture	
Minimal to absent: nondiagnosis	0
Intermediate: some preservation	1
Well preserved: diagnosed easily	2
Cellular degeneration	
Severe: nondiagnosis	0
Intermediate: diagnosis possible	1
Minimal: diagnosed easily	2

0-≤2 points: unable to diagnose; >2-≤5 points: diagnosable; >5-≤8 points: easy to diagnose.

**Table 2:** Cytopathological results of 240 cases of FNA and 240 cases of FNC.

Bethesda	No. of thyroid nodules (%)	
	FNA	FNC
i	3 (1.25)	3 (1.25)
ii	100 (41.67)	95 (39.58)
iii	0 (0)	4 (1.67)
iv	20 (8.33)	20 (8.33)
v	13 (5.42)	17 (70.83)
vi	104 (43.33)	101 (42.08)
Total	240 (100)	240 (100)

FNA: fine-needle aspiration; FNC: fine-needle capillary sampling.

**Table 3:** Specimen scores of 25-, 23-, and 22-gauge needles in FNA and FNC.

Scores	No. of thyroid nodules (%)							
	FNA				FNC			
	25 G	23 G	22 G	*P* value	25 G	23 G	22 G	*P* value
0-2 points	34 (14.17)	47 (19.58)	37 (15.42)	0.027	30 (12.50)	45 (18.75)	42 (17.50)	0.192
3-5 points	79 (32.92)	95 (39.58)	94 (39.17)		113 (47.08)	112 (46.67)	102 (42.50)	
6-8 points	127 (52.92)^a^	98 (40.83)^a^	109 (45.42)		97 (40.42)	83 (34.58)	96 (40.00)	

FNA: fine-needle aspiration; FNC: fine-needle capillary sampling. ^a^*P* = 0.008 (significant difference between 25 G and 23 G in FNA).

**Table 4:** Average score of each parameters in 22 G, 23 G, and 25 G groups of FNA and FNC.

Smear parameters	Thyroid nodules: mean score ± SD					
	FNA			FNC		
	25 G	23 G	22 G	25 G	23 G	22 G
Background of blood or clot	1.38 ± 0.72^a^	1.20 ± 0.74^a^	1.31 ± 0.70	1.28 ± 0.67^b^	1.15 ± 0.72^b^	1.23 ± 0.73
Number of obtained cells	1.39 ± 0.71^a^	1.23 ± 0.76^a^	1.32 ± 0.69	1.26 ± 0.66^b^	1.14 ± 0.75^b^	1.21 ± 0.72
Preserved tissue architecture	1.37 ± 0.72^a^	1.20 ± 0.74^a^	1.28 ± 0.72	1.30 ± 0.70^b^	1.15 ± 0.73^b^	1.22 ± 0.74
Cellular degeneration	1.36 ± 0.72^a^	1.19 ± 0.75^a^	1.28 ± 0.70	1.28 ± 0.61^b^	1.16 ± 0.71^b^	1.24 ± 0.71
Total score	5.50 ± 2.87^a^	4.82 ± 2.95^a^	5.19 ± 2.81	5.12 ± 2.69^b^	4.60 ± 2.90^b^	4.90 ± 2.90

FNA: fine-needle aspiration; FNC: fine-needle capillary sampling. ^a^*P* < 0.017 (significant difference between 25 G and 23 G in FNA). ^b^*P* < 0.017 (significant difference between 25 G and 23 G in FNC).

## Data Availability

Due to the privacy of patients involved in this clinical study, raw data access is limited. If you are interested in this study, you can consult the corresponding author through the email address to obtain the privacy hidden data.

## References

[1] Haugen B. R., Alexander E. K., Bible K. C. (2016). 2015 American Thyroid Association management guidelines for adult patients with thyroid nodules and differentiated thyroid cancer: the American Thyroid Association guidelines task force on thyroid nodules and differentiated thyroid cancer. *Thyroid*.

[2] Kim M. J., Kim E. K., Park S. I. (2008). Us-guided fine-needle aspiration of thyroid nodules: indications, techniques, results. *Radiographics*.

[3] Wu H. H., Jones J. N., Osman J. (2006). Fine-needle aspiration cytology of the thyroid: ten years experience in a community teaching hospital. *Diagnostic Cytopathology*.

[4] Sangalli G., Serio G., Zampatti C., Bellotti M., Lomuscio G. (2006). Fine needle aspiration cytology of the thyroid: a comparison of 5469 cytological and final histological diagnoses. *Cytopathology*.

[5] Song H., Wei C., Li D. (2015). Comparison of fine needle aspiration and fine needle nonaspiration cytology of thyroid nodules: a meta-analysis. *BioMed Research International*.

[6] Braun H., Walch C., Beham A., Moinfar F. (1997). Fine needle capillary cytology versus fine needle aspiration cytology--a comparison of quality between puncture techniques in the ENT area. *Laryngo- Rhino- Otologie*.

[7] Tangpricha V., Chen B. J., Swan N. C., Sweeney A. T., de las Morenas A., Safer J. D. (2001). Twenty-one-gauge needles provide more cellular samples than twenty-five-gauge needles in fine-needle aspiration biopsy of the thyroid but may not provide increased diagnostic accuracy. *Thyroid*.

[8] Gumus M., Cay N., Algin O. (2010). Comparison of 21 and 27 gauge needles for determining sample adequacy in the aspiration biopsy of thyroid nodules. *Diagnostic and Interventional Radiology*.

[9] Martin H. E., Ellis E. B. (1930). Biopsy by needle puncture and aspiration. *Annals of Surgery*.

[10] Zhou J. Q., Zhang J. W., Zhan W. W. (2014). Comparison of fine-needle aspiration and fine-needle capillary sampling of thyroid nodules: a prospective study with emphasis on the influence of nodule size. *Cancer Cytopathology*.

[11] Pacini F., Schlumberger M., Dralle H., Elisei R., Smit J. W. A., Wiersinga W. (2006). European consensus for the management of patients with differentiated thyroid carcinoma of the follicular epithelium. *European Journal of Endocrinology*.

[12] Haddadi-Nezhad S., Larijani B., Tavangar S. M., Nouraei S. M. (2003). Comparison of fine-needle-nonaspiration with fine-needle-aspiration technique in the cytologic studies of thyroid nodules. *Endocrine Pathology*.

[13] Abati A. (2008). The national cancer institute thyroid FNA state of the science conference: "wrapped up". *Diagnostic Cytopathology*.

[14] Baloch Z. W., Cibas E. S., Clark D. P. (2008). The national cancer institute thyroid fine needle aspiration state of the science conference: a summation. *CytoJournal*.

[15] Lee Y. J., Kim D. W., Shin G. W. (2019). Comparison of cytological adequacy and pain scale score in ultrasound-guided fine-needle aspiration of solid thyroid nodules for liquid-based cytology with 23- and 25-gauge needles: a single-center prospective study. *Scientific Reports*.

[16] Mair S., Dunbar F., Becker P. J., Du Plessis W. (1989). Fine needle cytology--is aspiration suction necessary? A study of 100 masses in various sites. *Acta Cytologica*.

[17] Romitelli F., Di Stasio E., Santoro C., Iozzino M., Orsini A., Cesareo R. (2009). A comparative study of fine needle aspiration and fine needle non-aspiration biopsy on suspected thyroid nodules. *Endocrine Pathology*.

[18] Degirmenci B., Haktanir A., Albayrak R. (2007). Sonographically guided fine-needle biopsy of thyroid nodules: the effects of nodule characteristics, sampling technique, and needle size on the adequacy of cytological material. *Clinical Radiology*.

[19] Tanaka A., Hirokawa M., Higuchi M. (2019). Optimal needle size for thyroid fine needle aspiration cytology. *Endocrine Journal*.

[20] Cerit M., Yucel C., Gocun P. U., Poyraz A., Cerit E. T., Taneri F. (2015). Ultrasound-guided thyroid nodule fine-needle biopsies--comparison of sample adequacy with different sampling techniques, different needle sizes, and with/without onsite cytological analysis. *Endokrynologia Polska*.

[21] Zhang L., Liu Y., Tan X., Liu X., Zhang H., Qian L. (2018). Comparison of different-gauge needles for fine-needle aspiration biopsy of thyroid nodules. *Journal of ultrasound in medicine*.

[22] Jung S. J., Kim D. W., Baek H. J. (2018). Comparison study of the adequacy and pain scale of ultrasound-guided fine-needle aspiration of solid thyroid nodules with a 21- or 23-gauge needle for liquid-based cytology: a single-center study. *Endocrine Pathology*.

